# Potential Role of CD47-Directed Bispecific Antibodies in Cancer Immunotherapy

**DOI:** 10.3389/fimmu.2021.686031

**Published:** 2021-07-08

**Authors:** Yan Yang, Zheng Yang, Yun Yang

**Affiliations:** ^1^ Department of Biochemistry and Molecular Biology, School of Basic Medical Sciences, Xinxiang Medical University, Xinxiang, China; ^2^ College of Public Health, School of Public Health, Zhengzhou University, Zhengzhou, China

**Keywords:** cancer immunotherapy, bispecific antibodies (bsAbs), CD47, immune checkpoint, macrophage, phagocytosis

## Abstract

The prosperity of immunological therapy for cancer has aroused enormous passion for exploiting the novel targets of cancer immunotherapy. After the approval of blinatumomab, a bispecific antibody (bsAb) targeting on CD19 for acute lymphoblastic leukemia, a few of CD47-targeted bsAbs for cancer immunotherapy, are currently in clinical research. In our review of CD47-targeted bsAbs, we described the fundamental of bsAbs. Then, we summarized the information of four undergoing phase I researches, reviewed the main toxicities relevant to CD47-targeted bsAb immunological therapy of on-target cytotoxicity to healthy cells and a remarkable antigen-sink. Finally, we described possible mechanisms of resistance to CD47-targeted bsAb therapy. More clinical researches are supposed to adequately confirm its security and efficacy in clinical practice.

## Introduction

Cancer is a serious health problem and the second primary cause of death all over the world. Data from the International Agency for Research on Cancer (IARC) displayed that there were about 19.2 million new cancer cases and 9.9 million new cancer deaths in 2020. Over the past few decades, the introduction of cancer immunotherapies, aiming to ameliorate anti-tumor immune responses with less off-target effects, compared with chemotherapies and other agents which straightway kill tumor cells, has significantly improved the survival outcome of patients (1–3). Immunotherapeutic agents are designed to activate or modulate innate or adaptive immune system to assault cancer cells *via* repairing or enhancing natural mechanisms, plenty of which are escaped or damaged in the development of disease, thereby inhibiting tumor growth and metastasis (4–6). Results from the KEYNOTE-024 study on patients with advanced non-small cell lung cancer revealed that the overall survival (OS) rate after five years was twice as high for patients that were treated with Keytruda (31.9%), compared to a chemotherapy regimen (16.3%) (7). Thus, immunotherapy is universally acknowledged to treat, and to heal, several kinds of cancer.

However, monotherapy with these and other drugs based on monoclonal antibodies cannot heal certain types of cancer, especially owing to T lymphocytes not actively participating in destroying tumors, nonetheless monoclonal antibodies merely restrain the combination of growth factors with the corresponding receptors. Monoclonal antibody interdicting the inhibitory signals which defend cancers from immune cells display fantastic outcomes while treating certain especial kinds of cancers. However, antibodies binding to two or more antigens (bsAbs), together with conjugated agents are research hotspots for chemo and radiotherapies.

## Therapeutic BsAbs in Oncology

The original concept of bispecific antibody (bsAb) was proposed 50 years ago by Nisonoff and co-workers (8). Subsequently, the utilization of bsAbs to redirect immune cells to kill tumor cells was then certified in the 1980s, which provided a promising immunotherapeutic approach for cancer therapy and diagnosis (9). Blinatumomab (Blincyto^®^, CD3 × B lymphocyte antigen CD19) was the first bsAb to be ratified by the US Food and Drug Administration (FDA) for the treatment of relapsed or refractory acute lymphoblastic leukemia (AML) in 2014 (10, 11). The remarkable success of blinatumomab has prompted pharmaceutical companies to efficiently generate and produce stable bsAbs. Till date, more than 100 bsAbs with different backbones and over 30 technology platforms have been reported and reviewed (12–16), and approximately, two-thirds of these bsAb therapeutics in the clinical pipeline are designated for the treatment in oncology (17), which either recruit and redirect the immune effector cells to kill cancer cells or interdict diverse signaling pathways *via* restraint of the ligand or the receptor (17–19).

**Figure 1:**
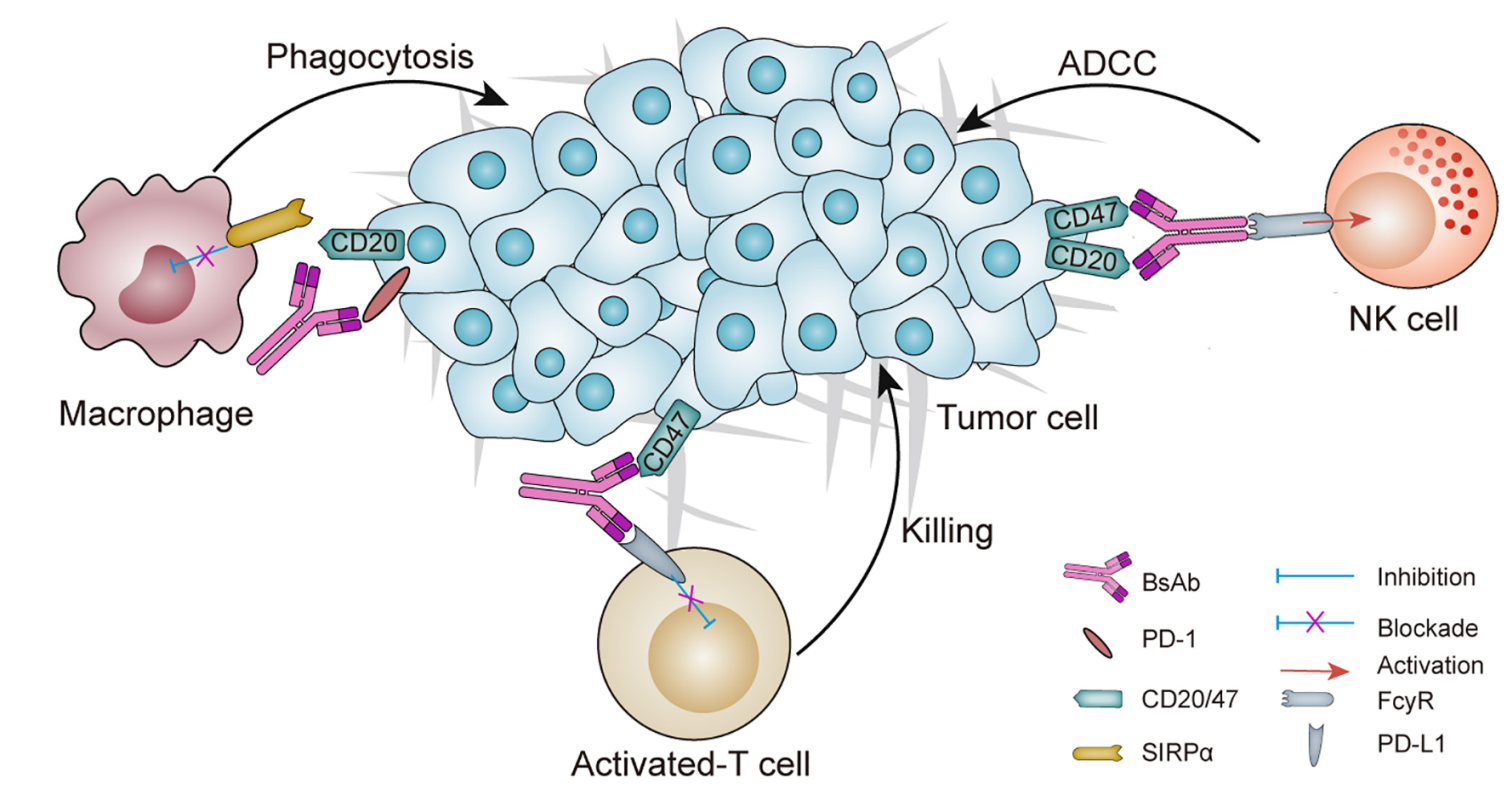
Schematic overview of a BsAb in cancer: mechanisms of action and potential targets. Dual checkpoints blockade is achieved by two-checkpoint blockers integrated into one antibody to inhibit two immune checkpoints simultaneously. Tumor targeted immunomodulators are designed for binding to one tumor-associated antigen (TAA) to inhibit TAA signaling pathway and the other immunomodulating receptor (e.g., PD-L1, CD47), thus regulating the immune system to attack the tumors. ADCC, antibody-dependent cell-mediated cytotoxicity; bsAb, bispecific antibody; NK, natural killer; SIRPα, signal-regulatory protein α.

BsAbs are genetically designed recombinant antibodies, which contain two disparate binding domains that allow simultaneous coupling with two diverse antigens or two diverse epitopes of the same antigen (**Figure 1**) (20, 21). Two diverse receptors or ligands on the surface of the same cell might be concurrently targeted by bsAbs, and they will give rise to restraint or stimulation of two diverse signaling pathways. Fusion of anti-tumor binding domain with the fragment crystallizable region (Fc) or the anti-CD3 binding domain is a conventional strategy to generate bsAbs, which has a great potential to recruit immune cells.

**Figure 2:**
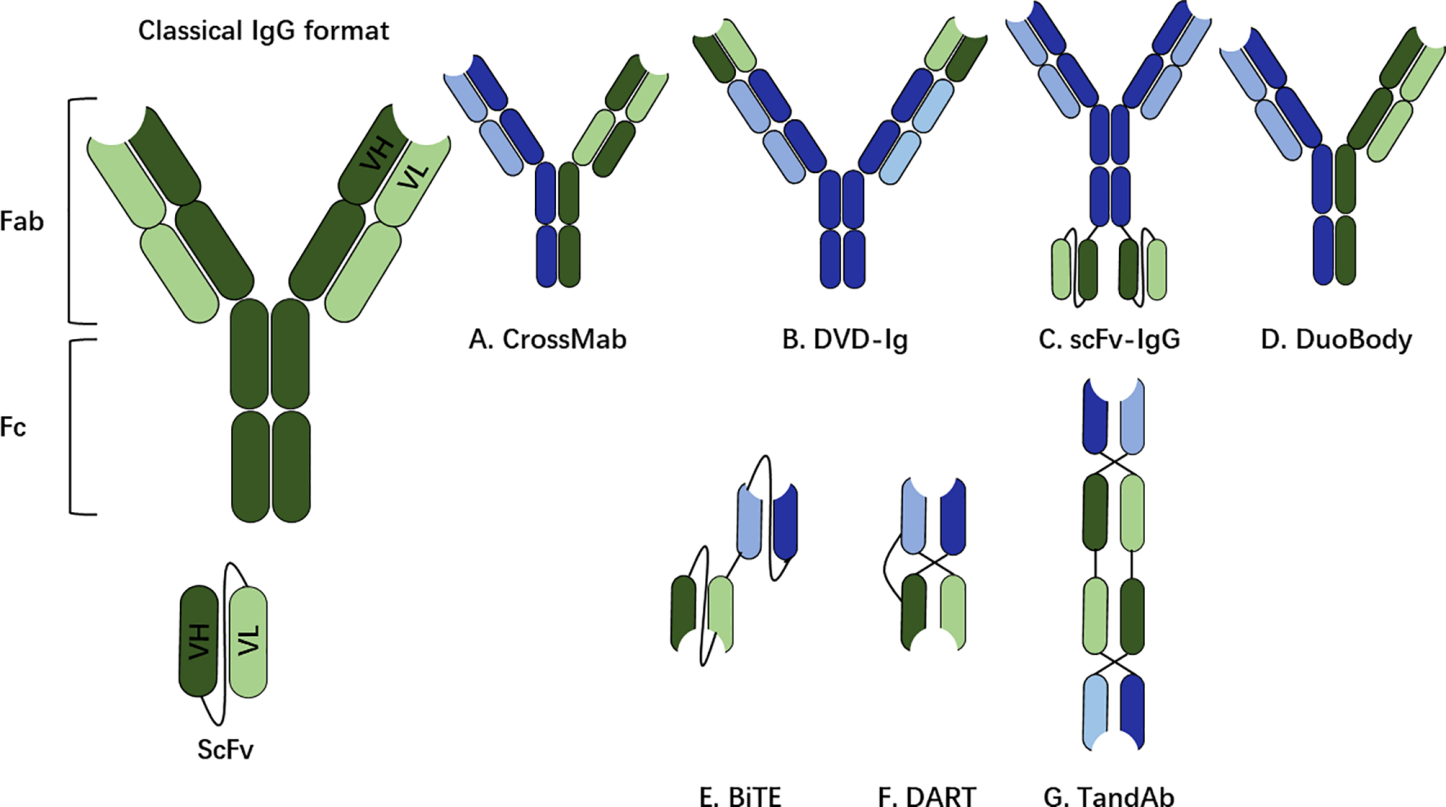
The non-IgG-like format and IgG-like format. The classical structure of a human immunoglobulin G (IgG) for reference, including fragment crystallizable (Fc) region and fragment antigen-binding (Fab). The binding part of the Fab region is called the single chain variable fragment (scFv), which is a synthetic protein composed of the heavy (VH) and light (VL) chains connected by a short peptide linger. **(A–D)** IgG-like bsAb formats (with Fc region). **(E–G)** non-IgG-like bsAb fragments (without Fc region). CrossMab Cross monoclonal antibody, DVD-Ig, dual-variable-domain immunoglobulin; scFv, single-chain variable fragment; BiTE, bispecific T-cell engager; DART, dual-affinity retargeting molecule; TandAb, tandem diabody.

The bsAbs can be categorized into two different formats: a non-IgG-like format and an IgG-like format (**Figure 2**), and the structural elements of each format have implications for engagement and mobilization of the immune system. Among the clinically approved bsAbs, Catumaxomab and Hemlibra (emicizumabkxwh) have the IgG-like format, while Blinatumomab has the non-IgG-like format.

The IgG-like bsAbs include the Fc region, which is an important determinant for bsAb in stimulating immune responses. Biochemical properties including affinity, glycosylation and isotype of the Fc region are responsible to trigger different immune effector cells. The Fc region triggers bsAb-mediated functionalities, including antibody-dependent cell-mediated cytotoxicity (ADCC), complement-dependent cytotoxicity (CDC) and antibody-dependent cellular phagocytosis (ADCP) (22).

Unlike the IgG-like bsAbs, the non-IgG-like bsAb format lacks the Fc region and in consequence, the molecular mass (30–50 kDa) is smaller than that of a whole IgG (150 kDa). Because the structure of a non-IgG-like format includes only VH and VL domains or the Fab fragment, such bsAb format depends only on Ag binding capacity for its therapeutic function. A major limitation of the non-IgG like bsAbs is their short-half life.

In contrast to antibody-drug conjugates and naked monoclonal antibodies (mAb), bsAbs can be engineered to recruit diverse effector immune cells through coupling with the membrane-associated antigen expressed from cancer cells, and the second antigen expressed in special immune cells, such as natural killer (NK) cells, effector T cells (23) or immunomodulatory proteins such as cluster of differentiation 47 (CD47) or programmed death receptors 1 (PD-1) (24). The superior effect might be ascribed to the tumor-directing function generated by bsAbs (25). Additionally, bsAbs execute their therapeutic effect by binding to receptors expressed on cell surface, without requiring for receptor internalization, which permits to target a disparate population of tumor antigens. A few of immunotherapies aiming to CD47, such as antibody-drug conjugates, chimeric antigen receptor (CAR) T cells and bsAbs, are now undergoing assessed in clinical trials or preclinical studies. We have recently reported our experience of constructing a novel bsAb fusion protein both targeting EGFR and CD47, which could reduce the ‘‘off-target’’ effects because of CD47 expression on red blood cells (RBCs). Our study demonstrated that bi-SP with modified treatment indices may have a potential to treat CD47+ and EGFR+ cancers in the clinic (26).

## Clinical Experience of CD47 as a Target for Cancer Immunotherapy

**Figure 3:**
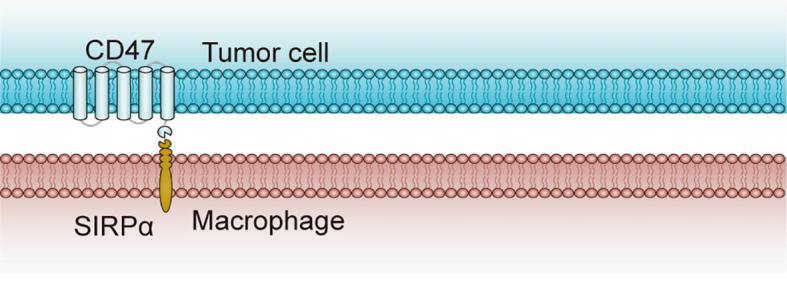
Structure and interaction of CD47 and SIRPα. CD47 contains one N-terminal extracellular IgV-like domain and five-membrane-spanning segments at the membrane. SIRPα, as the CD47 ligand, contains three extracellular IgSF domains, one transmembrane spanning region and an intracellular domain with ITIM motifs. CD47-SIRPα binding prevents the host cell from being targeted for phagocytosis, while anti-CD47 antibodies can block the suppression signal and promote phagocytosis by macrophages (27). CD47, cluster of differentiation 47; ITIM, immunoreceptor tyrosine-based inhibitory motif; SIRPα, signal-regulatory protein α.

CD47 [Integrin-associated protein (IAP)] has a molecular weight of 45-55 kDa (**Figure 3**) with five-membrane-spanning segments in the membrane, which coprecipitates with platelet-derived β3 integrin and placental αvβ3 integrin (28). As we know, it is the receptor of thrombospondin family members, and is an extraordinary member of the membrane protein immunoglobulin (Ig) superfamily with a single extracellular IgV-like region (29). CD47 is expressed at the plasma membrane of almost all cell types including mesenchymal stromal cells and blood cells, and is known to mediate vascular smooth cell proliferation and migration (30), platelet activation and spread (31), and recruit granulocytes and T cells to infections positions (32, 33). It was initially introduced as a tumor antigen involved in ovarian cancer, and multiple studies have indicated that CD47 is generally upregulated in various types of malignancies (34), such as myeloma (35), leiomyosarcoma (36), leukemia (37), non-Hodgkin’s lymphoma (38), breast cancer (39), osteosarcoma (40), and hepatocellular carcinoma (41). It may be a mechanism of cancer immune evasion, and this mechanism-driving overexpression in tumor cells seems to be a compensatory response to a pro-inflammatory tumor microenvironment with high levels of TNF-α (42), which activates NF-κB signaling and thus regulates CD47 expression (43). Therefore, the CD47/SIRPα axis is considered as a novel existing target in immunology (39), and CD47 will have a major role in tumorigenesis, since its enhanced expression in cancer cells enables evasion of phagocytosis (44).

Notably, CD47 sends “don’t eat me” signals by inhibiting phagocytosis of tumor cells and triggering an immune evasion and therefore serves as a myeloid-specific immune checkpoint when CD47 binds to signal regulatory protein α (SIRPα) that is an inhibitory receptor on macrophages and dendritic cells. In view of the interaction between CD47 and SIRPα in tumor cells restricts the anti-tumor immune response, treatment with anti-CD47 antibodies that restrain CD47 signaling in tumor cells was able to induce the phagocytosis of tumor cells by macrophages and stimulate anti-tumor immune responses *in vivo* (45). Consequently, a number of CD47-targeting antibodies are in clinical trials of numerous cancers spanning both hematological malignancies and solid tumors.

The most prominent clinically approved CD47-targeted immunotherapeutic is the humanized anti-CD47 antibody (Hu5F9-G4) that targets CD47 to induce phagocytosis, which was developed at Stanford University. Hu5F9-G4 is currently being assessed in phase I clinical research of patients with solid malignancies (www.clinical trials.gov identifier: NCT02216409). Also, HU5F9-G4 can trigger an effective anti-tumor T cell response, which cross-presents cancer cell antigens *via* macrophages, in order to provide protection against tumor recurrence.

A variety of ways have been established to target the CD47 pathway, such as directly interdicting of CD47 or its macrophage receptor SIRPα. Therefore, interdiction of CD47 in preclinical studies utilizing monospecific antibodies has shown promising effects. Nevertheless, the application of anti-CD47 antibodies still faces some safety risk issues. First, CD47 is ubiquitously expressed at the membranes of all cells, including RBCs, under normal and pathological conditions, generating cytotoxicity and antibody depletion. Therefore, comparing to traditional cancer immunotherapeutics, bsAbs targets CD47 along with other tumor antigens as a viable strategy for directing the synergistic benefits of combination therapy specifically toward tumor cells, has a potential to enhance security and efficacy. Secondly, anti-CD47 antibody monotherapy cannot completely get rid of lymphoma. In contrast, combination stratagems which induce adoptive immunity or refer to the utilization of anti-CD20 antibody, macrophage agonists, including IFN-c, interleukin-10, and other agents (e.g., caspase modulators and F-actin regulators), might possess enduring and efficient anti-lymphoma effects. Thirdly, the effects of diverse ways of interdicting CD47, including anti-CD47 antibodies or scFv derived from an antibody, are still uncharted.

## BsAb Targeting CD47 and Other Targets That Are Currently in Clinical Trials in Oncology

BsAbs co-targeting CD47 and other tumor-specific antigens may improve the binding specificity of CD47-directed antibody, thus enhancing safety and efficacy. Considering these above, bsAbs targeting tumor-specific receptors possessing high binding affinity on one arm and CD47 with lower affinity on the other arm, are nowadays being assessment in clinical trials or are in preclinical study. There are currently four bsAbs constructs targeting CD47 for the treatment of patients with various kinds of cancers, which are undergoing assessed in the clinics at present (**Table 1**).

**Table 1:** CD47-targeted bsAbs in clinical trials as of March 2021 (44).

bsAb	Sponsor	Format	Targets	Disease area	Development stages	ClinicalTrials.gov Identifier	Status	Preliminary Clinical Data Reported (Ref.)
IBI322	Innovent Biologics (Suzhou) Co. Ltd.	Not available	CD47/PD-L1	Solid tumors and hematological tumors	Phase I	NCT04338659	Not yet Recruiting	No
					Phase I	NCT04328831	Recruiting	Yes (25, 46)
HX009	Waterstone Hanxbio Pty Ltd	Antibody-receptor fusion	PD-1/CD47	Malignancies (liver cancer, stomach cancer, and colorectal cancer and so on)	Phase I	NCT04097769	Recruiting	No
IMM0306	ImmuneOnco	Antibody-receptor fusion	CD47/CD20	B-cell Non-Hodgkin’s Lymphoma	Phase I	NCT04746131	Not yet Recruiting	No
TG-1801	NovImmune, TG Therapeutics	Antibody-receptor fusion	CD47/CD19	Haematologicalmalignancies (B cell lymphoma)	Phase I	NCT03804996	Recruiting	No
SL-172154	Shattuck Labs, Inc	Antibody-receptor fusion	SIRPα-Fc-CD40L	Ovarian Cancer	Phase I	NCT04406623	Recruiting	Yes (47)

### IBI322

IBI322 is the first anti-CD47/PD-L1 checkpoint bsAb that inhibits both the PD-1/PD-L1 and CD47/SIRP-α pathways, which is used for the treatment of patients with advanced malignancies (25). Preclinical studies have demonstrated that IBI322 can effectively block CD47/SIRP-α interaction and induce macrophages to phagocytose CD47-expressed tumor cells, which is equivalent to anti-CD47 monoclonal antibody. And it was suggested that the affinity of IBI322 to PD-L1 was stronger than that to CD47, implying a potential therapy regime in PD-L1-positive cancers (48). IBI322 also effectively blocks the binding of PD-1 to PD-L1 and activates CD4+T lymphocytes, which is comparable to anti-PD-L1 monoclonal antibody. Since PD-L1 is expressed in tumor cells, IBI322 can selectively bind to tumor cells more effectively than anti-CD47 monospecific antibody, thus reducing the possibility of binding to CD47 expressed on RBCs, which could ultimately reduce the toxicity associated with anti-CD47 antibodies. Therefore, IBI322 has stronger anti-tumor activity and higher safety profile.

The first case had been triumphantly administrated in a Phase I clinical trial (CIBI322A101) of the promisingly first-in-class recombinant anti-CD47/PD-L1 bsAb (IBI322) in China. CIBI322A101 is a Phase Ia/Ib clinical study conducted in China to evaluate IBI322 in the treatment of patients with advanced malignancies. Meanwhile, dose selection for first-in-class human (FIH) studies of IBI322, authorized by the National Medical Products Administration (NMPA) for clinical trials (IND No. CXSL1900125) was evaluated, suggesting that the preliminary pharmacodynamics (PD) study with 0.34 mg/kg was rational (46). For scientific confirmation, it still needs more researches at different doses to be conducted in future.

Anti-CD47 antibodies have the tendency to attack normal cells. However, IBI-322 is preferentially distributed in PD-L1-positive tumor cells, thus decreasing the possible adverse effects of this target associated with monospecific anti-CD47 antibodies (25). In addition, bispecific monoclonal antibody enhances cytotoxicity, antibody selectivity and functional affinity by targeting effector cells directly to tumor cells. Preliminary results showed that IBI322 had higher efficacy *in vivo*, tumor-rich distribution and better safety than the mono-specific anti-CD47 antibody (25). Bispecific antibody could offer a lower-cost solution for patients compared with two monoclonal antibody combinatorial therapies. Therefore, the development of the anti-CD47/PD-L1 bsAb will provide patients with a novel, comprehensive, effective, and cost-saving treatment regimen. IBI322 has the potential to benefit more patients in need. Given that IBI322 is currently undergoing a phase I dose escalation trials in China (NCT04328831) and USA (NCT04338659), there is no clinical data reported for it. In China, CIBI322A101 is a Phase 1a/1b clinical study conducted to evaluate IBI322 in the treatment of patients with advanced malignancies. The Phase 1b study will be carried out to evaluate the efficacy of IBI322 in lung, cervical, esophageal, head and neck squamous cell and liver carcinomas. Collectively, more clinical studies are needed still in the clinic.

### HX009

As crucial innate and adaptive immune checkpoints on cancer cells, CD47 and PD-L1 coordinate to inhibit immune sensing. Preclinical studies have demonstrated a bsAb co-targeting PD-L1 and CD47 (49) dramatically improved tumor targeting and treatment outcome *vs*. monotherapy *in vitro* and *in vivo*. Anti-PD-1 antibodies restored large amounts of exhausted T cells and anti-CD47 antibodies stimulated phagocytosis of macrophage. Based on this, the anti-PD-1/CD47 bsAb, HX009, was developed by Hangzhou Hanx Biopharmaceutics, Inc. (HanxBio), to treat patients with advanced solid tumors, including gastric cancer, colorectal cancer and liver cancer. Consisting of human IgG4-Fc region of anti-PD-1 mAb and extracellular domain (ECD) of SIRPα, it achieves the synergistic anti-tumor effects by simultaneously activating both innate and acquired immune responses to suppress tumor immune escape and release immune suppression at immune checkpoints.

This antibody-receptor fusion format may have time-cost savings by adapting the natural receptor to the bsAb over the two mAbs-based bsAb for which one more mAb has to be developed. However, due to relatively lower stability of the receptor portion, the antibody-receptor fusion proteins might suffer less stability than the bsAbs based on two mAbs. Currently, HX009 is in early phase I clinical trial (NCT04097769) for patients with advanced solid tumors. So far, no clinical data has been reported for safety and efficacy of HX009.

### IMM0306

IMM0306, developed by Shanghai ImmuneOnco Biopharmaceuticals Co. (ImmuneOnco), is a bispecific recombinant antibody-receptor fusion protein. It is designed to target both CD47 and CD20 on B cells but avoid binding to human RBCs, which could simultaneously act on the tumor disease targets and modulate the immune system. By activating the phagocytosis potency of macrophages and triggering antigen-specific T cells *via* tumor antigen presentation, IMM0306 will become a new hot point in the research of cancer immunotherapy in future (50).

Extensive characterization *in vitro* demonstrated that IMM0306 binds to both CD47 and CD20 with affinity 3-8 folds lower than either single-targeted molecule. However, it has greater pro-phagocytosis activity over CD47-positive target cells, and even stronger ADCC activity than Rituximab (an anti-CD20 mAb). Intriguingly, IMM0306 has no binding activity at all toward human RBCs, albeit much lower binding activity toward monkey RBCs (51). Treatment of tumor-implanted SCID mice with IMM0306 significantly inhibited tumor growth and led to eradication of the tumor cells from 5 out of 8 mice, which is much more effective than Rituximab (51). Besides, the *in vivo* studies demonstrated that IMM0306 did not bind to human RBCs and did not induce T cell apoptosis. It can clear lymphoma at a low dose (1.5 mg/kg) (52), showing obvious advantages in safety and clinical development. Preclinical study in non-human primates demonstrated a favorable pharmacokinetic profile with no obvious hemotoxicity following single as well as multiple administrations at different dosage.

All these studies above suggest that antibody-trap like IMM0306 might be an ideal approach for CD47-targeted immunotherapy development since selective avoidance of RBCs mediated antigen-sink as well as anemia could be achieved along with the robust anti-tumor activity. In addition, the preclinical studies confirmed that IMM0306 achieved significant therapeutic effects in various tumor models. IMM0306 is now being assessed in phase I trial (NCT04746131) for the evaluation of safety and effect in patients with B-cell non-Hodgkin’s lymphoma. Meanwhile, dose selection of IMM0306, authorized by NMPA for clinical trials (IND No. CTR20192612), is being evaluated for patients with refractory or recurrent CD20 positive B-cell non-Hodgkin’s lymphoma. Until now, there is no clinical data reported for IMM0306.

### TG-1801

As the first-in-class anti-CD47/CD19 bsAb, TG-1801 is a fully humanized IgG1, targeting the ‘don’t eat me’ self-defense signal which defends cancer cells against the immune system. This bsAb is designed by combining a low-affinity CD47 targeting antibody with a high-affinity antibody against CD19, to make sure that CD47 is employed by the bsAb only on tumor cells that co-expressed both antigens. It has an enhanced Fc-mediated phagocytosis and reserved its activity while the existence of high levels of non-tumor-associated CD47 (53). TG-1801 is designed to selectively target CD47 on CD19+ B cells, sparing RBCs and platelets, with the blockade of CD47-SIRPα macrophage checkpoint on mature B cells. It will avoid off-target toxicity, representing a novel immunological therapeutic strategy with potential for synergistic or complimentary activity with drugs in the current pipeline. In addition, the co-targeting of CD47 and CD19 enhances the expected safety, as well as induces antibody dependent cytotoxicity (ADCC) by retaining the function of its IgG1 Fc region, thereby providing a second mechanism for anti-tumor activity.

To conclude, TG-1801 might play a crucial part in improving the outcome of patients with B cell malignancies. Meanwhile, TG Therapeutics first demonstrated the synergistic effect of TG-1801 in combination with ublituximab (anti-CD20 monoclonal antibody) and umbralisib (PI3K-δ/casein kinase-1ϵ inhibitor) (54), and the synergistic tumor growth inhibition appeared to be mediated by increased infiltration of immune effector cells (55). At present, TG-1801 is undergoing assessment in Phase I trial (NCT03804996) to evaluate its safety and efficacy for treating patients with B cell lymphoma. Thus far, there is no clinical data reported for TG-1801.

### SL-172154

SL-172154, developed by Shattuck Labs Inc., is a novel fusion protein consisting of human SIRPα and CD40L (SIRPα-Fc-CD40L) linked *via* a human Fc. It is designed to block the CD47 immune checkpoint while simultaneously activating the CD40 pathway through the dual mechanism, which are both checkpoint blockade and TNF activation. Being one of bifunctional fusion protein candidates, SL-172154 is developed through Shattuck Agonist Redirected Checkpoint (ARC) Platform Technology, which is designed to solve structural limitations. Previous studies found that SL-172154 significantly improved rejection of both primary and secondary tumors, as compared with individual antibodies targeting CD40 and CD47 used alone or in combination, similar to PD-1-Fc-OX40L (56). Notably, the safety and efficacy of the bsAb in nonhuman primates *in vivo* that, the bsAb stimulated dose-dependent elevation in multiple serum cytokines and CD40^+^ B-cell margination in cynomolgus macaques, without causing hemolysis or thrombocytopenia, provides justification to further explore this strategy in patients with cancers (47).

Currently, SL-172154 is being evaluated in a phase 1 trial (NCT04406623) for patients with ovarian cancer. So far, no clinical data has been reported for safety and efficacy of SL-172154.

## Biosafety Concerns and Future Directions Associated With CD47-Targeted BsAbs

Given the huge potential of anti-CD47 bsAbs, there is ongoing interest in expanding this field in cancer immunotherapy and several bsAbs targeting CD47 have entered into clinical trials. So far in current phase 1 studies, blast reduction and complete remission rates have been observed, although safe targeting doses to be used in future phase 2 clinical trials still need to be determined for each bsAb targeting CD47 construct. Despite ubiquitously expressed on almost all cell types, on-target cytotoxicity of CD47 to healthy cells and a prominent antigen-sink phenomenon have influenced the development of CD47-targeting agents (57). Therefore, it is important to improve our understanding for potential biosafety problems.

Since CD47 is ubiquitously expressed, potential problems with anti-CD47 antibodies as anticancer agents were possible off-target effects such as anemia (58). CD47 is a crucial regulator of RBCs turnover (59). Buatois et al. (60) indicated that Hu47F9-G4 alone or in combination with other antibodies may cause accidental killing of normal RBCs, potentially resulting in anemia. Hence, there are concerns that CD47-targeting antibodies would expedite RBCs clearance and cause hemolytic anemia since several agents in this class have learn about RBC affinity while others do not. For CD47-targeting bsAbs in preclinical research, various methods are being used to attempt to alleviate this on-target cytotoxicity. For example, the NHP study indicated that a second dose of IBI322 (25) resulted in an extra less drop in the RBCs and hematocrit indices. While RBC counts of both groups began to revert on day 11, IBI322 treatment resulted in a dramatically enhanced RBC count, compared with the Hu5F9 group on day 15. Given that the cross-reaction of IBI322 with cyno PD-L1 and CD47 at approximate affinities to receptors, it is rational to expect that IBI322 could decrease CD47 target-mediated side-effect in patients. CD47 might become an efficient target of this stratagem soon afterward. Comprehensive assessment of IBI322 for the safety and efficacy is necessary in future.

Notably, CD47-targeting agents (i.e., Hu5F9-G4 and TTI-621) could cause acute anemia and thrombocytopenia in people (61–63), which might as well as depend upon the Fc format. The toxicity of anti-CD47 antibodies appears to be Fc-dependent, given that anti-CD47 antibodies, and SIRPα-Fc fusion proteins give rise to this toxicity, while high-affinity SIRPα monomers do not (39, 64, 65). These findings suggest that further studies should aim to optimize the structure of the anti-CD47 bsAbs when attempting to design novel drugs without unwanted side-effects. How to reduce or avoid damage to normal cells while exerting antitumor effects is one of the problems that needs to be considered when designing anti-CD47 therapeutics.

In addition, the expression of CD47 on normal tissues may create an ‘antigen sink’ that prevents anti-CD47 therapeutic antibodies from reaching tumor cell targets *in vivo*, which also pose a problem in the development of anti-CD47 bsAbs. One strategy to circumvent this issue is by reducing the bsAbs affinity for CD47 but retaining the ability to block the CD47-SIRPα interaction and elevating the affinity to a second tumor antigen. In future studies, more strategies by targeting CD47 and its ligands specifically on tumor cells should be investigated.

Upon macrophage or dendritic cell-mediated phagocytosis of cancer cells by CD47 blockade, these phagocytes may present tumor antigens to T cells to induce anti-tumor T cell responses (66, 67). Therefore, the regimen by combining with T-cell checkpoint inhibitors (with anti-PD-1 or PD-L1 agents) may further augment T cell responses and enhance efficacy. Several pre-clinical studies have demonstrated that CD47 blockade in combination with T cell checkpoint inhibitors can enhance antitumor activity (68–70). Importantly, CD47-targeting agents have the potential to augment existing antibody therapies by adding the benefit of blocking the CD47-mediated inhibitory signal to the established therapeutic effect of pro-phagocytic antitumor antibodies.

We should realize that future improvements in cancer screening and precision medicine will enable the identification and stratification of specific tumor types and/or stages of cancer that would be most amenable to treatment with a certain type or types of anti-CD47 treatment.

## Conclusion

Recently, we have witnessed much progress with bsAb technologies and therapeutics as reviewed here and elsewhere (13, 71), which have found wide applicability to immunotherapy for cancer treatment. And thus, many bsAb constructs with differing mechanisms of action are being investigated, each with their own limitations and advantages. In this review, we summarized diverse strategies of cancer immunotherapy and characterized the breakthrough in bsAbs development which might be devoted into enhancing the treatment effect and decreasing untoward effects, which mark the beginning of a new era for cancer immunotherapy. Although cancer immunotherapy is rapidly advancing, the effectiveness of bsAbs, especially CD47 bsAbs, can vary between different cancer types, different studies, and even different cancer types, in other words, the therapeutic efficacy of anti-CD47 agents may be tumor specific. Thus, the exploration of novel strategies by targeting macrophage checkpoints is under development. In addition to CD47/SIRPα-targeting antibodies and fusion proteins, the other potential CD47-directed strategies should also be considered.

In summary, two exclusion conditions must be set in the anti-CD47 bsAbs screening process: 1) RBC binding and erythrocyte agglutination induction; 2) Induction of T lymphocyte apoptosis. Without the exclusion of these above, it will be difficult to achieve clinical success. At present, most of the clinical trials targeting CD47 are in phase I clinical trials, which mainly focus on hematoma, thus its value in the solid tumors has yet to be verified. In addition, IgG4 antibodies against CD47 do not respond well to solid tumors, even when used in combination with other antibody drugs. Rigorously, pre-clinical assessments of such treatments are warranted. Based on innovative techniques, we expect that CD47 bsAbs discussed in our review will be more extensively and innovatively designed for immunological therapy, therefore facilitating their effectiveness, as well as decreasing immunologically relevant untoward effects. Future research will be required to raise our comprehension of tumor immunology, which will provide important insights into developing more bsAbs.

## Author Contributions

YY (1^st^ author) wrote the original draft. ZY and YY (3^rd^ author) revised the manuscript. YY (1^st^ author) prepared tables and figures. YY (3^rd^ author) reviewed and edited. All authors contributed to the article and approved the submitted version.

## Funding

This work was supported by the National Natural Science Foundation of China (No. 81703054), Natural Science Foundation of Henan (No. 202300410322), and Training Plan for Young Backbone Teachers in Universities of Henan Province (No. 2020GGJS143).

## Conflict of Interest

The authors declare that the research was conducted in the absence of any commercial or financial relationships that could be construed as a potential conflict of interest.
